# Renoprotective effects of renin–angiotensin system inhibitor combined with calcium channel blocker or diuretic in hypertensive patients: A PRISMA-compliant meta-analysis

**DOI:** 10.1097/MD.0000000000004167

**Published:** 2016-07-18

**Authors:** Yiming Cheng, Rongshuang Huang, Sehee Kim, Yuliang Zhao, Yi Li, Ping Fu

**Affiliations:** a Renal Division, Department of Internal Medicine, West China Hospital of Sichuan University, Sichuan; b Department of Biostatistics, University of Michigan School of Public Health; c Kidney Epidemiology and Cost Center, University of Michigan, MI; d West China Biostatistics and Cost-Benefit Analysis Center West China Hospital of Sichuan University, Chengdu, Sichuan, China.

**Keywords:** calcium channel blocker, diuretic, hypertension, renin–angiotensin system inhibitor, renoprotective effects

## Abstract

**Objectives:**

To conduct a meta-analysis of studies comparing the renoprotective effects of angiotensin-converting enzyme inhibitor/angiotensin receptor blocker (ACEI/ARB) combined with either calcium channel blocker (CCB) or diuretic, but not both, in hypertensive patients.

**Data sources:**

Pubmed, Embase, Medline, and Cochrane databases were searched to identify randomized controlled trials (RCTs) of blood pressure lowering treatments in patients with hypertension.

**Study selection:**

RCTs comparing the renoprotective effects of ACEI/ARB plus CCB with ACEI/ARB plus diuretic in hypertensive patients, with at least one of the following reported outcomes: urinary protein, estimated glomerular filtration rate/creatinine clearance (eGFR/CrCl), or serum creatinine.

**Results:**

Based on 14 RCTs with 18,125 patients, statistically significant benefits were found in ACEI/ARB plus CCB for maintaining eGFR/CrCl (standardized mean difference [SMD] = 0.36; 95% confidence interval [CI]: 0.20–0.53; *P* < 0.001), serum creatinine reduction (mean difference [MD] = −0.05 mg/dL; 95% CI: −0.07 to −0.03; *P* < 0.001). However, no statistical differences were found between the 2 therapeutic strategies in terms of urinary protein (MD = 7.48%; 95% CI: –6.13% to 21.08%; *P* = 0.28; *I*^2^ = 92%).

**Conclusions:**

This meta-analysis concluded that ACEI/ARB plus CCB have a stronger effect on the maintenance of renal function in patients with hypertension than ACEI/ARB plus diuretic.

## 1 Introduction

Hypertension that is not controlled can lead to kidney damage.^[1]^ Although blood pressure targets vary slightly depending on comorbid conditions (diabetes and chronic kidney disease [CKD]),^[2–4]^ maintaining blood pressure below the given target is crucial. Moreover, urinary protein control in hypertensive patients is also required to slow the progression of kidney disease and cardiovascular damage.^[5,6]^

To control blood pressure and urinary protein levels, studies have shown that the majority of hypertensive patients need at least 2 agents.^[2–4]^ In particular, when a renin–angiotensin–aldosterone system (RAAS) inhibitor is used, combining this type of medication with a calcium channel blocker (CCB) or a thiazide diuretic improves patients’ prognosis.^[2–4]^ The combination of an RAAS inhibitor and a CCB has been recommended since CCBs have shown potent antihypertensive and cardiovascular protective effect,^[2–4,7]^ while having minimal effects on metabolism,^[8,9]^ thus decreasing blood pressure safely and synergistically.^[10]^ In contrast, the combination of an RAAS inhibitor and a diuretic has been recommended as diuretics reduce plasma volume and cardiac output.^[11]^

The synergistic effects of these 2 different types of combined treatment have received much attention, thus multiple clinical trials have been conducted to directly compare their benefits and adverse effects.^[12–25]^ However, the results have been inconsistent, and therefore inconclusive, in part due to differences in study populations, sample size, and/or different comorbidities (e.g., diabetes, CKD). What is needed, therefore, is a systematic review of the existing studies. In the present review, the meta-analysis technique was used to compare the renoprotective effects between angiotensin-converting enzyme inhibitor/angiotensin receptor blocker (ACEI/ARB) plus CCB and ACEI/ARB plus diuretic in patients who are under hypertension treatment.

## 2 Methods

### 2.1 Data source and search strategy

Publications were identified by searching electronic databases including PubMed, Embase, Medline, and the Cochrane Library from the earliest available date of indexing to October 2015. Search terms included hypertension, ACEIs, angiotensin receptor antagonists, CCBs, diuretics, combination therapy, proteinuria, serum creatinine, and kidney function. In addition to these terms, some similar expressions for “combination therapy” were used, such as “combined treatment.” Some pharmacological names for medication, such as “dipeptidyl carboxypeptidase inhibitor” for “angiotensin-converting enzyme inhibitors” to widen the search.

### 2.2 Study selection

Studies were included if they were randomized controlled trials (RCTs), regardless of parallel or cross-over design, that compared ACEI/ARB plus CCB with ACEI/ARB plus diuretic in hypertensive patients. The use of combination therapy was defined as simultaneous treatment of either an ACEI/ARB plus a CCB or an ACEI/ARB plus a diuretic in hypertensive patients. Diagnostic criteria used to define hypertension were: systolic blood pressure of at least 140 mmHg, and/or diastolic blood pressure of at least 90 mmHg.^[3]^ This meta-analysis only included studies with at least one of the following laboratory measurements: change in estimated glomerular filtration rate/creatinine clearance (eGFR/CrCl), change in serum creatinine, and change in a urinary protein-related item. The urinary protein-related item was defined as any of the following measures: urinary albumin to creatinine ratio (ACR), urinary protein to creatinine ratio, urinary albumin excretion (UAE), 24-hour total urinary protein, or 24-hour urinary albumin. The information on the eGFR/CrCl, serum creatinine, and urinary protein related item was required since these biomarkers were used as surrogates for renal effect.^[5,26–28]^

### 2.3 Outcome measures

This meta-analysis aimed to evaluate the renoprotective effects of combining ACEI/ARB with either CCB or diuretic. It has been shown that eGFR/CrCl and serum creatinine are indicators reflecting renal function,^[27]^ while eGFR/CrCl and urinary protein are important biomarkers that predict renal damage progression to some extent.^[5,26,28]^ Therefore, as the outcomes of interest, changes in eGFR/CrCl, serum creatinine, and urinary protein from baseline were considered.

### 2.4 Data extraction and quality assessment

The process of studies identification, data extraction, analyses conduction, and results reporting were performed following the steps listed in the Preferred Reporting Items for Systematic Reviews and Meta-Analyses, a guideline for systematic reviews, and meta-analyses of health care interventions. The Preferred Reporting Items for Systematic Reviews and Meta-Analyses is a concise checklist consisting of 27 items deemed essential for reporting a clear and completed systematic review.^[29]^ Two authors (YC and RH) independently reviewed the data, analyzed the types of studies, and assessed the eligibility and methodological quality of the articles included in this meta-analysis. Any discrepancies were resolved by consensus.

The extracted data consisted of 3 components: study characteristics, patient characteristics, and outcomes. The study characteristics included name of the 1st author, publication date, sample size, follow-up period, and interventions (type, dose, and duration of therapy). The patient characteristics included demographic factors (age, sex, and race) and clinical factors at baseline (eGFR/CrCl, serum creatinine, urinary protein-related items, systolic blood pressure, and diastolic blood pressure).

The methodological quality of RCTs were assessed using Jadad Scale.^[30]^ The Jadad Scale is an assessment score based on the degree of participant randomization, blinding, and the report of withdrawals and dropouts. A higher score indicates better quality. The risk of bias in each included study was assessed using the Cochrane Collaboration tool.^[31]^ This tool addresses 6 specific domains: sequence generation, allocation concealment, blinding of subjects/outcome assessors, incomplete outcome data, selective outcome reporting, and other issues. In each RCT, every domain was assessed to be high or low risk of bias, or unclear. An overall assessment of each RCT was graded as low risk if all the domains were assessed as low risk of bias, or at most 2 domains were assessed to be unclear (while the rest of domains were at low risk). Otherwise, an overall assessment of the RCT was graded as high risk of bias.

### 2.5 Statistical analysis

The intervention of interest was ACEI/ARB plus CCB versus ACEI/ARB plus diuretic. The *I*^2^ statistical index was used to assess heterogeneity across the studies, and *I*^2^ > 50% was considered as an indication of high heterogeneity.^[32]^ A random effects model was applied to combine the studies since statistical heterogeneity existed in the treatment effects of some outcome measures.^[33]^ For all continuous outcomes, mean differences (MDs) were used when the unit of measurement was consistent across studies, while standardized mean differences (SMDs) were used with a mixed-unit of measurement.^[34]^ The 95% confidence intervals (CIs) were reported. *P*-values less than 0.05 were considered statistically significant, except for the test of heterogeneity where *P* < 0.1 was used. All statistical analyses were carried out using RevMan statistical software version 5.3.

### 2.6 Sensitivity analyses and subgroup analyses

To evaluate robustness of the meta-analysis results, we carried out 2 sensitivity analyses: high quality studies versus low quality studies, and studies with small sample size versus large sample size.

Subgroup analyses were conducted to explore possible sources of heterogeneity and clinical significance related to the following characteristics: race; lab indices in urinary protein: urinary ACR, UAE, urinary protein to creatinine ratio, 24-hour total urinary protein, and 24-hour urinary albumin; and comorbid conditions: diabetes and CKD.

### 2.7 Ethical approval

Patient informed consent and ethical approval were not necessary because all analyses were conducted on the basis of previous information.

## 3 Results

### 3.1 Study characteristics

**Figure 1:**
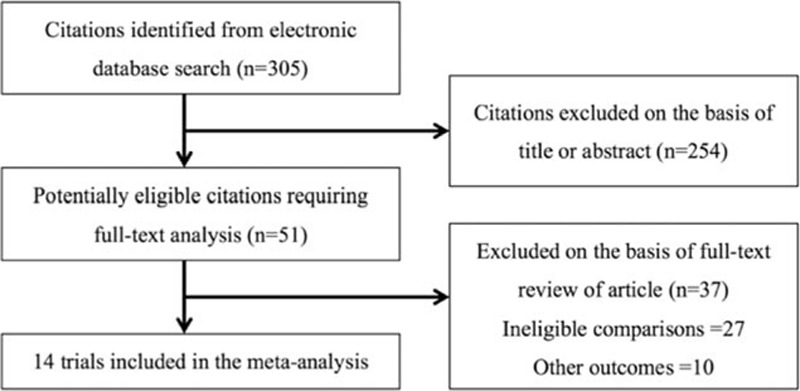
Flow diagram for the study selection.

A total of 305 studies were identified, among which 254 studies were excluded by title and abstract review. By full paper review, an additional 37 studies were excluded since the studies did not meet inclusion criteria for either intervention or outcome measures. Finally, there were 14 studies that compared the renoprotective effect of the combination of ACEI/ARB plus CCB to that of the combination of ACEI/ARB plus diuretic during 3 to 40 months of follow-up,^[12–25]^ and hence included in this meta-analysis (Fig. 1). One study was based on a cross-over RCT,^[24]^ and 13 studies were based on parallel RCTs. The final 14 RCTs consisted of a total of 18,125 patients with hypertension. Among them, 8 RCTs recruited Asian subjects only,^[13–15,18,20,21,23,24]^ while the other 6 RCTs were conducted in either mixed-race or unmentioned-race populations. Six RCTs compared the 2 combined treatments in 1001 patients with diabetes mellitus,^[13,14,16,17,22,25]^ and 3 RCTs compared treatments in 1079 CKD patients.^[19,23,24]^ Regarding the sample size, ACCOMPLISH (11,506 patients) and COLM (5141 patients) were the 2 largest RCTs.^[19,20]^

The assessment of quality and risk of bias are summarized in Table 1. Five studies obtained Jadad scores lower than 3,^[15,16,18,20,24]^ indicating low quality. The Cochrane Collaboration assessment suggested that 8 studies were at a high risk of bias^[12,14–16,18,20,23,24]^ (Table 1). The main reason for the high risk was the lack of detailed descriptions of concealing, randomization, or allocation.

**Table 1:**
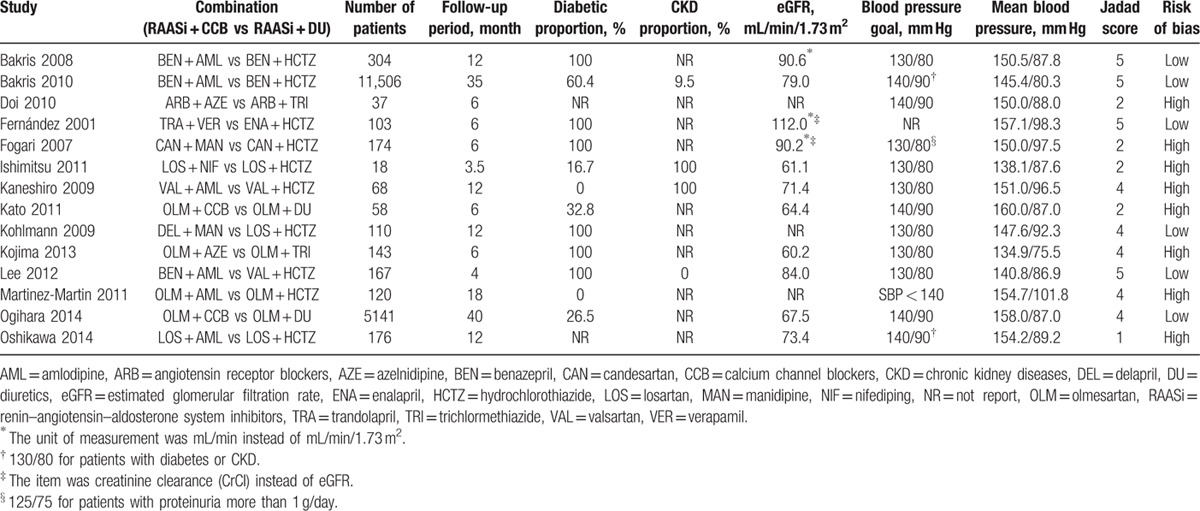
Characteristics of randomized controlled trials included in this meta-analysis.

### 3.2 Effect on eGFR/CrCl

**Figure 2:**
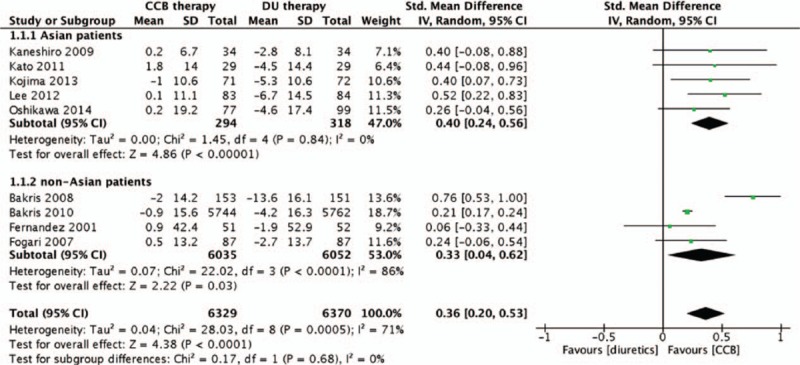
Comparison of ACEI/ARB plus CCB therapy with ACEI/ARB plus diuretic therapy for the changes of eGFR/CrCl. ACEI = angiotensin-converting enzyme inhibitor, ARB = angiotensin receptor blocker, CCB = calcium channel blocker, CrCl = creatinine clearance, eGFR = estimated glomerular filtration rate.

Seven trials reported the change in eGFR^[13–15,19,20,23,25]^ and 2 trials reported the change in CrCl.^[16,17]^ Four showed that ACEI/ARB plus CCB was the better treatment choice than ACEI/ARB plus diuretic,^[13,14,19,25]^ whereas the remaining 5 studies showed no difference between the 2 treatment strategies. In this meta-analysis, the endpoints of eGFR and CrCl were synthesized in 1 forest plot as both were considered as indicators of kidney function,^[27,35,36]^ representing the capability for material filtration of kidneys.^[37]^ Since some studies used mL/min^[16,17,25]^ and the others used mL/min/1.73 m^2^ as the unit of measurement, the SMD was used for this outcome.^[34]^ The pooled analysis showed a better effect of ACEI/ARB plus CCB in maintaining eGFR/CrCl, compared to ACEI/ARB plus diuretic (SMD = 0.36; 95% CI: 0.20–0.53; *P* < 0.001; *I*^2^ = 71%; Fig. 2). Note that it is not desirable to compare eGFR between 2 different racial populations because of racial differences in muscle mass that cause different concentrations of serum creatinine, an important parameter to determine eGFR/CrCl.^[38]^ Therefore, subgroup analysis was conducted with Asian patients. The subgroup analysis result was consistent with that in the mixed-race populations, except that there existed no heterogeneity in the pooled effects; *I*^2^ decreased from 71% to 0% (Fig. 2).

### 3.3 Effect on serum creatinine

**Figure 3:**
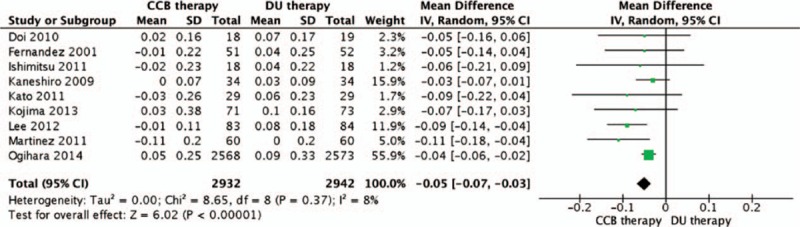
Comparison of ACEI/ARB plus CCB therapy with ACEI/ARB plus diuretic therapy for the changes of serum creatinine. ACEI = angiotensin-converting enzyme inhibitor, ARB = angiotensin receptor blocker, CCB = calcium channel blocker.

Nine trials assessed the effects of ACEI/ARB plus CCB and ACEI/ARB plus diuretic on serum creatinine.^[12–15,17,18,21,23,24]^ Three reported that ACEI/ARB plus CCB showed a statistically significant difference in serum creatinine reduction compared to ACEI/ARB plus diuretic,^[12,13,21]^ which was consistent with the meta-analysis result (MD = −0.05 mg/dL; 95% CI: −0.07 to −0.03; *P* < 0.001; *I*^2^ = 8%; Fig. 3). Moreover, sensitivity and subgroup analyses yielded consistent results showing a better renoprotective effect of ACEI/ARB plus CCB than ACEI/ARB plus diuretic.

### 3.4 Effects on urinary protein

**Figure 4:**
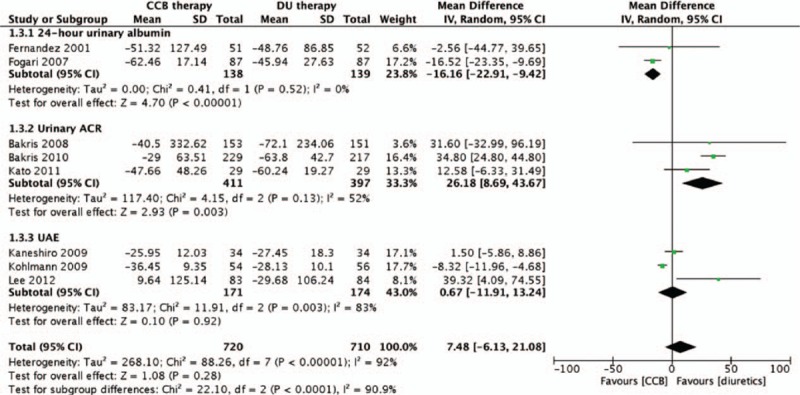
Comparison of ACEI/ARB plus CCB therapy with ACEI/ARB plus diuretic therapy for the changes of urinary protein related items. ACEI = angiotensin-converting enzyme inhibitor, ARB = angiotensin receptor blocker, CCB = calcium channel blocker.

To assess renoprotective effects, 2 studies used 24-hour urinary albumin,^[16,17]^ 3 studies used urinary ACR,^[15,19,25]^ and 3 studies used UAE.^[13,22,23]^ After integrating the outcomes of 24-hour urinary albumin, urinary ACR, and UAE, the meta-analysis showed that ACEI/ARB plus diuretic resulted in a 7.48% larger decline in the pooled urinary outcome, although the decline was not statistically significant (MD = 7.48%; 95% CI: −6.13% to 21.08%; *P* = 0.28; *I*^2^ = 92%; Fig. 4). However, for sensitivity analysis of 1 large sample-size study, ACCOMPLISH, results showed ACEI/ARB plus diuretic therapy reduced more urinary protein than ACEI/ARB plus CCB therapy and this difference was statistically significant (MD = 34.84%; 95% CI: 24.80%–44.80%).

## 4 Discussion

To the best of our knowledge, this is the 1st meta-analysis for exploring renoprotective effects between 2 combination therapies, ACEI/ARB plus CCB and ACEI/ARB plus diuretic. This meta-analysis showed a significantly better effect of ACEI/ARB plus CCB therapy on maintaining eGFR/CrCl and reducing serum creatinine, compared to ACEI/ARB plus diuretic. However, this meta-analysis was unable to show statistical differences in control of urinary protein. This is partly because of the mixed items related to urinary protein (24-hour urinary albumin, UAE, and urinary ACR) and low concentration of urinary protein. For the latter reason, many subjects of the meta-analysis had a diagnosis of general hypertension or early stage of diabetes with a concentration of urinary protein in the normal or slightly microalbuminuria range.^[39,40]^ However, eGFR/CrCl and serum creatinine are stronger and more accurate markers of kidney function, particularly in early stage of renal disease.^[39,40]^

Although the exact mechanism between CKD and hypertension has not been very clear, a gradually accepted view points out that kidneys contribute to and are damaged by hypertension both pathophysiologically and clinically.^[1,41,42]^ On the one hand, a decreasing glomerular filtration rate will activate the sympathetic and/or RAASs and result in refractory hypertension;^[42]^ on the other hand, the uncontrolled hypertension will cause glomerular injury and result in a gradual loss of kidney function in patients suffering from general hypertension^[1,41]^ or with comorbidities, such as CKD^[43]^ and diabetic mellitus.^[44]^ To control blood pressure and attenuate kidney damage, the strategy of blood pressure control becomes an advisable and feasible method to break the infernal circle. In recent 10 years, American, European, and Japanese guidelines have put forward and revised some recommendations in the profile of blood pressure control for renal protection.^[2–4,45–47]^ The goals of blood pressure control in the current guidelines become not that strict as the previous due to limited efficacy and increase of adverse events with high dose of antihypertensive agents. However, the recommendations of combination therapy remain the same. These guidelines recommend utilizing combination therapies including ACEI/ARB plus CCB and ACEI/ARB plus diuretic. In the present studies, surrogate biomarkers (eGFR/CrCl, serum creatinine, and urinary protein) were used to assess renoprotective effects of the combined treatments. Although it is often necessary to use surrogate markers for clinical endpoints, limitations exist in that the actual clinical evidence such as doubling of serum creatinine, progression to dialysis, and death are not directly considered. There was 1 trial included, ACCOMPLISH, investigating the risk of progression of CKD or death, and they found a lower risk of renal events in ACEI/ARB plus CCB group, compared to ACEI/ARB plus diuretic group (HR = 0.73; 95% CI: 0.64–0.84; *P* < 0.001). This meta-analysis, integrating ACCOMPLISH study with 13 other trials, has shown a consistent conclusion of better efficacy of ACEI/ARB plus CCB using the 2 different surrogate biomarkers: eGFR/CrCl and serum creatinine.

Different studies used different units of measurement to report the eGFR/CrCl. Processing mixed types of data and mixed units of measurement will increase the risk of bias and thus become an inevitable limitation in meta-analyses. A strength of this meta-analysis is that the mixed-unit of measurement has been taken into account by using an SMD.^[34]^ SMD is the ratio of MD to the pooled standard deviation, making the magnitude of variation more comparable. A larger MD between the 2 treatment groups and (or) a smaller standard deviation will result in a bigger absolute value of SMD. For example, an SMD of 0.36 with a positive value implies that the improvement in eGFR/CrCl was larger in ACEI/ARB plus CCB group, compared to ACEI/ARB plus diuretic group, with an increment approximately one-third the pooled standard deviation.

Speaking of limitations in this meta-analysis, they have been stated and analyzed in Section 3 and the former part of Section 4. In summary, the limitations include the heterogeneous race of populations, the mixed units of data, and the lack of actual clinical evidence. All the limitations had an impact on the source of bias, which has been overcome, in part, through conducting additional and extensive sensitivity and subgroup analyses, focusing powerful and accurate biomarkers (eGFR/CrCl and serum creatinine) and using SMD in statistical process.

To evaluate renoprotective effects as the primary endpoints, a large observational study is ongoing at West China Hospital of Sichuan University. The study population is patients diagnosed as CKD and using ACEIs, ARBs, CCBs, α-blockers, β-blockers, and diuretics as antihypertensive agents. With no aforementioned issues, the renoprotective effects of these agents will be assessed based on renal events including doubling of serum creatinine and progression to dialysis, as well as the surrogate biomarkers considered in this study.

## 5 Conclusion

This meta-analysis included RCTs to assess the effect of ACEI/ARB plus CCB on kidney-related outcomes in patients with hypertension compared to ACEI/ARB plus diuretic. In particular, for maintaining eGFR and reducing serum creatinine, better effects of ACEI/ARB plus CCB treatment have been shown, compared to the combination treatment of ACEI/ARB plus diuretic. No consistent evidence was shown for the urinary protein control. Hence, additional large and high-quality prospective studies are needed to assess urinary protein and a more direct effect on clinical endpoints such as end-stage renal disease or mortality.
